# Height, weight and body mass index of children with congenital cataracts before surgical treatment

**DOI:** 10.1186/s12886-017-0513-4

**Published:** 2017-07-11

**Authors:** Jingjing Chen, Duoru Lin, Zhuoling Lin, Xiaoyan Li, Qianzhong Cao, Zhenzhen Liu, Erping Long, Xiaohang Wu, Li Zhang, Xiaojia Zhou, Lisha Wang, Jing Li, Jun Fu, Haotian Lin, Weirong Chen, Yizhi Liu

**Affiliations:** State Key Laboratory of Ophthalmology, Zhongshan Ophthalmic Center, Sun Yat-sen University, Guangzhou, Guangdong 510060 People’s Republic of China

**Keywords:** Congenital cataracts, Height, Weight, Body mass index

## Abstract

**Background:**

To investigate the distribution of the height, weight and body mass index (BMI) of children with congenital cataracts (CC) before surgical treatment.

**Methods:**

This prospective study included children with CC ≤14 years of age presenting at the Zhongshan Ophthalmic Center from Jan. 2013 to Aug. 2016. The height, weight, and BMI measurements of all participating children were obtained and compared with the World Health Organization Child Growth Reference (WHO Reference), matched by age and gender. The presence of a family history of CC or complicated systemic diseases as well as parental education levels and family income were also recorded.

**Results:**

In total, 595 children with CC were included. The mean age was 52.75 ± 33.99 months, and 34.29% (204/595) of them were unilateral cases. Among all of the children, 6.72% (40/595) of cases were complicated by systemic diseases. More than 1/5 (21.01%, 125/595) of the children had a family history of CC and exhibited bilateral involvement. Less than 1/4 (23.2) of the mothers were highly educated, and more than half of the families had a family income below the city average. Height, weight, and BMI measurements of most children with CC were within the normal ranges (±95% CI of the WHO Reference). Compared to the WHO Reference, both girls and boys aged 2–5 years revealed shorter heights, and the girls aged 5–14 years exhibited a shorter height, lower body weight and lower BMI. The heights of the children with CC and systemic diseases were also shorter than the WHO Reference. The children with CC who had a family history of disease had shorter heights and lower BMIs than children with CC but no family history, and the measurements of both groups were lower than the WHO Reference values.

**Conclusions:**

The height, weight and BMI of most of the children with CC in this study were within the normal ranges of the WHO Reference. However, the children with CC and concomitant systemic diseases and those with a family history of CC had shorter heights and lower BMIs. This information aids in our understanding of the physical development of children with CC.

## Background

Congenital cataracts (CC) has been listed as one of the leading causes of childhood blindness in recent years [1]. Although CC is a rare disease with a relatively low prevalence of 4.24/10,000 [2, 3], the visual function of children with CC is severely affected. The accumulated evidence indicates that CC is a genetically hereditary disease [4], and a family history of CC is confirmed through pedigree and genetic analyses in nearly one-third of children with CC [5]. However, the degree to which the development of the height and weight of children with CC, the most important indicators of childhood physical development and health [6], are affected by this hereditary, systemic disease is unknown. Nearly all of the relevant published reports have focused on CC diagnosis and treatment, rather than physical development [7]. Thus, the present study, supported by the Zhongshan Ophthalmic Center (ZOC) [8], which is the largest eye care facility in Guangzhou City in southern China, analyzed the distribution of the height, weight, and body mass index (BMI) of children with CC. To our knowledge, this study is the first to focus on the physical development of children with CC and may provide a useful reference for national management strategies for diagnosing and treating CC.

## Methods

### Patients and ethics statement

This prospective study included children with CC ≤14 years of age presenting at the ZOC from Jan. 1, 2013, to Aug. 1, 2016. Participants were eligible if the children were newly diagnosed with CC by two independent, experienced pediatric ophthalmologists (HTL &WRC) before setting up files in the CC database for them. Information regarding concomitant systemic diseases and family histories of CC of each child was carefully gathered and recorded based on medical records, routine physical examinations, abnormal appearances, and parental statements. Children from welfare homes and those with unclear family histories or systemic disease information were excluded. This study was approved by the Human Research Ethics Committee of the ZOC, Sun Yat-sen University. All procedures adhered to the tenets of the Declaration of Helsinki, and written informed consent was obtained from at least one parent of each CC child.

### Measurements of physical development

The height and weight of each CC child were measured in centimeters (cm) and kilograms (kg) (accurate to one decimal place), and the BMI was calculated based on the height and weight measurements (BMI = weight [kg]/height [m]^2^). According to the WHO Child Growth Reference (WHO Reference), the height/length of children aged less than 2 years should be measured lying down for an accurate BMI calculation. The height of children older than 2 years was measured while they were standing up with bare feet, and their body weight was measured with a scale (RGZ-120-RT, Heng Qi Chang, Wuxi, China) while the patients were wearing a thin dress in a warm room. All children were measured by one experienced examiner (ZLL), and the measurement readings were confirmed by another two independent researchers (JJC & XYL). The children too young to actively cooperate were measured after routine pre-examination sedation with 10% chloral hydrate (0.8 ml/kg, oral or rectal administration) [9]. The height and weight of the children were measured 3 times, and the mean of 2 stable measurements was calculated for each parameter.

To further analyze the physical development of the children with CC, this study also compared the CC measurements with the WHO Reference values for height, weight, and BMI at every month of age. The WHO Reference, published in 2007, is available at “http://www.who.int/childgrowth/standards/en/” and was downloaded on Sept. 2016. The growth reference data for the height/length, weight, and BMI of children with ages ranging from birth to 14 years are included in the WHO Reference.

### Statistical analysis

Statistical analysis was performed using the Statistical Package for the Social Sciences (SPSS version 19.0, Chicago, Illinois, USA). Absolute frequency (n) and relative frequency (%) were used to analyze the number of children with CC, their gender, and the proportions of children exhibiting complications involving systemic diseases or family histories of CC. The mean and standard deviation (mean ± SD) were used for the analysis of height, weight, and BMI measurements. The Kolmogorov-Smirnov test was used to evaluate the normality of the distribution for all variables. The physical development of the children with CC complicated by systemic diseases or family histories of CC was compared with that of the remaining children with CC by matching ages and genders and then using the paired t test. For further comparison with the WHO Reference, the height, weight, and BMI measurements of the children with CC were averaged at every month of age and compared with the WHO Reference by month of age using paired t tests. A *P*-value of <0.05 was considered statistically significant.

## Results

**Fig. 1:**
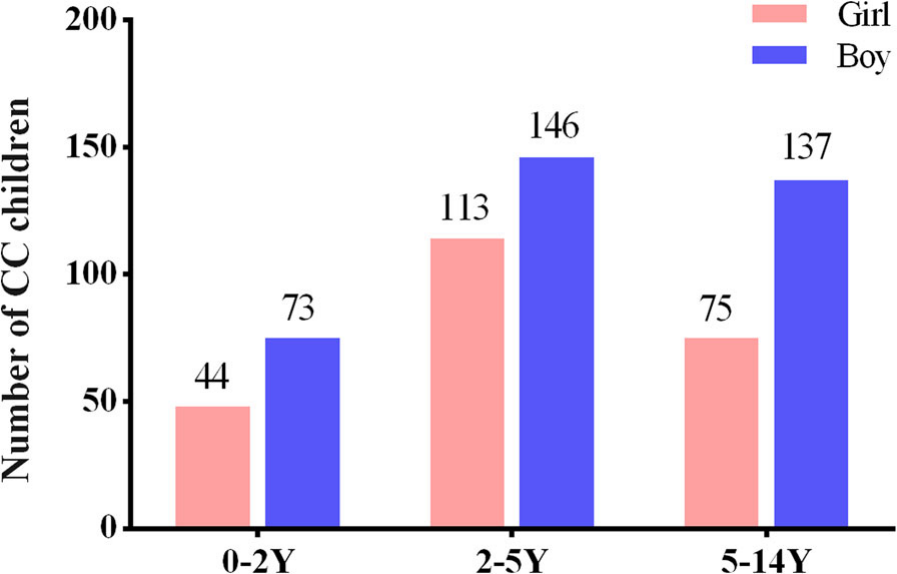
Distribution of children with CC stratified by different genders and ages. CC: congenital cataract; Y: years

**Fig. 2:**
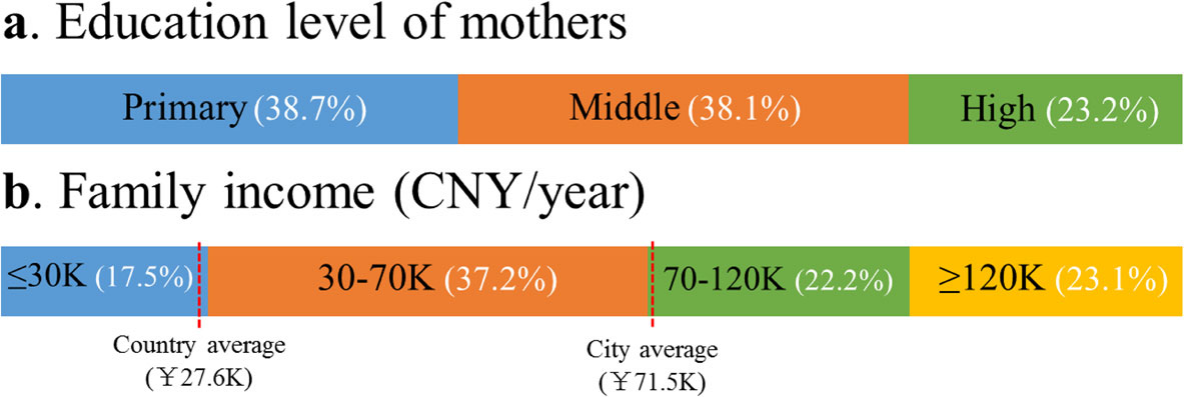
Distribution of the mother’s education level and family income among children with CC. **a** Only 23.2% of the mothers were highly educated. **b** More than half of the CC families (54.7%) had a family income below the city average. CNY: China Yuan

In total, 595 children with CC were included, consisting of 358 boys and 237 girls (Fig. 1). The mean age was 52.75 ± 33.99 months, and 34.29% (204/595) of them were unilateral cases. The mean height, weight, and BMI were 89.61 ± 18.37 cm, 13.50 ± 2.85 kg, and 16.17 ± 3.25 kg/m^2^, respectively. Among all of the children in this study, 6.72% (40/595) were complicated with systemic diseases, including 8 cases of favism and 5 cases with congenital heart diseases. More than 1/5 (21.01%, 125/595) of the children had a family history of CC and bilateral involvement. As shown in Fig. 2, less than 1/4 (23.2%) of the mothers were highly educated, and more than half of the CC families (54.7%) had a family income below the annual city average (CNY 71.5 K in 2011).

**Fig. 3:**
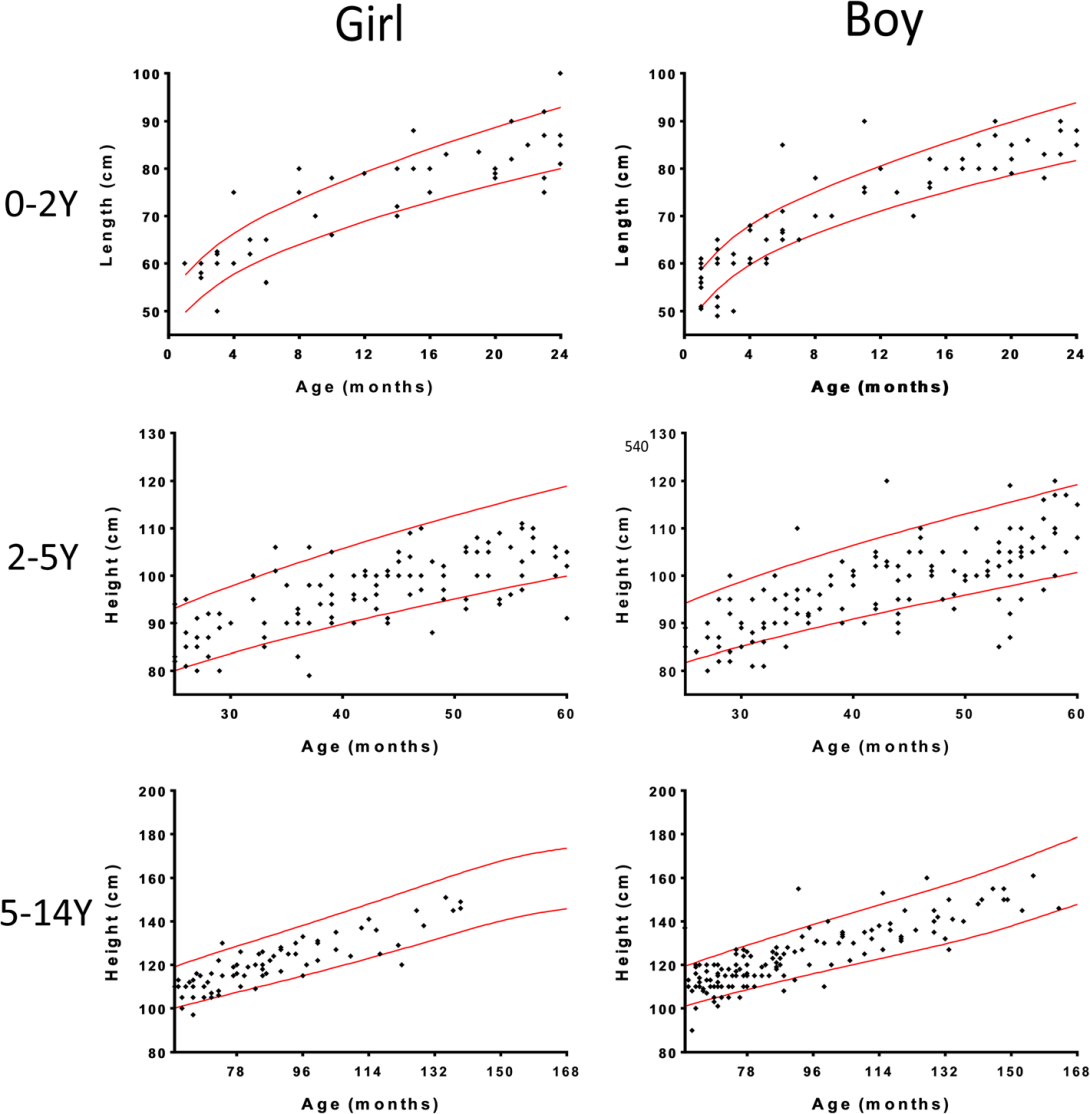
Distribution of the height measurements of all children with CC by gender and age. Most height measurements were within the normal range (red line: ±95% CI of the WHO Reference). CC: congenital cataract; Y: years

**Fig. 4:**
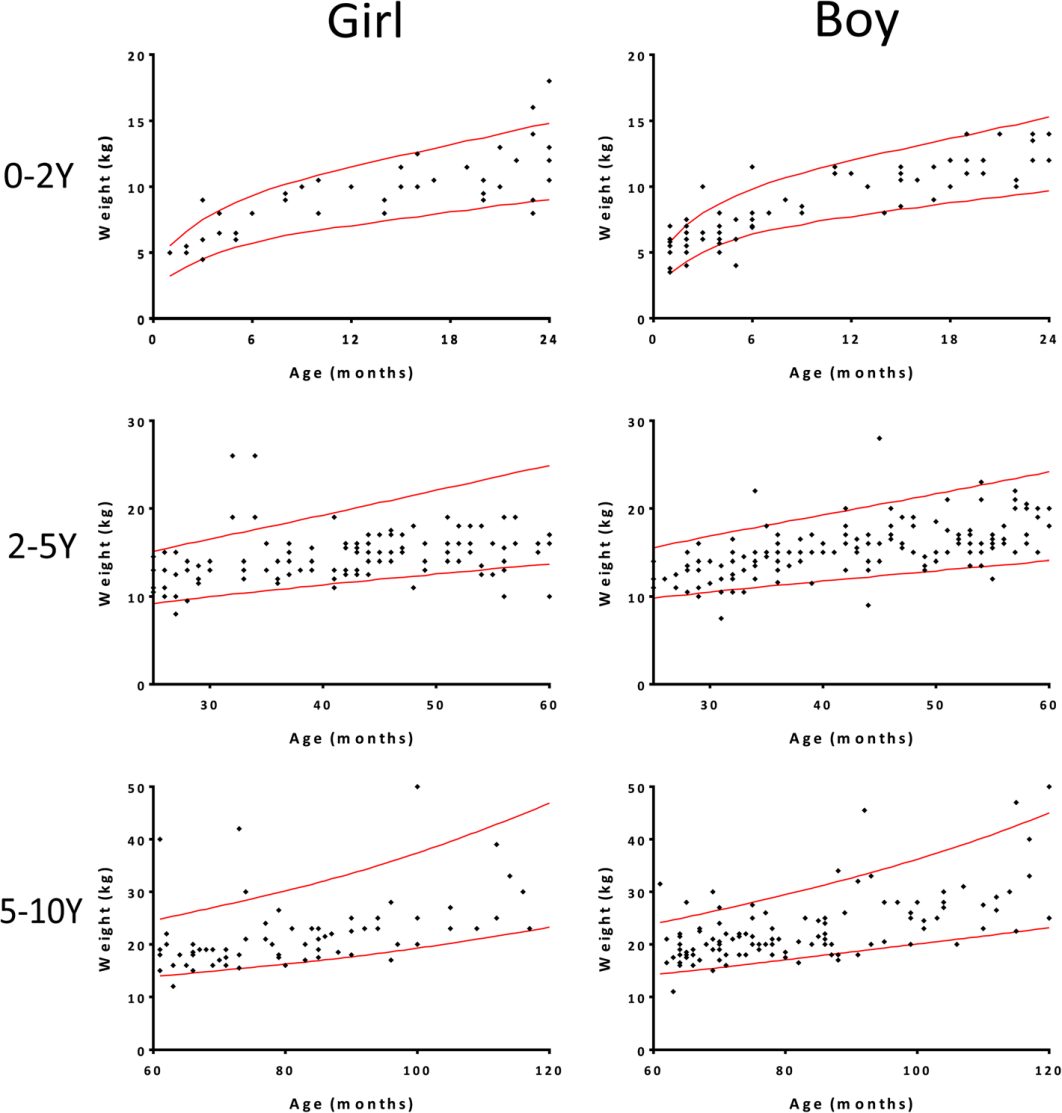
Distribution of the weight measurements of all children with CC by gender and age. Most weight measurements were within the normal range (red line: ±95% CI of the WHO Reference). CC: congenital cataract; Y: years

**Fig. 5:**
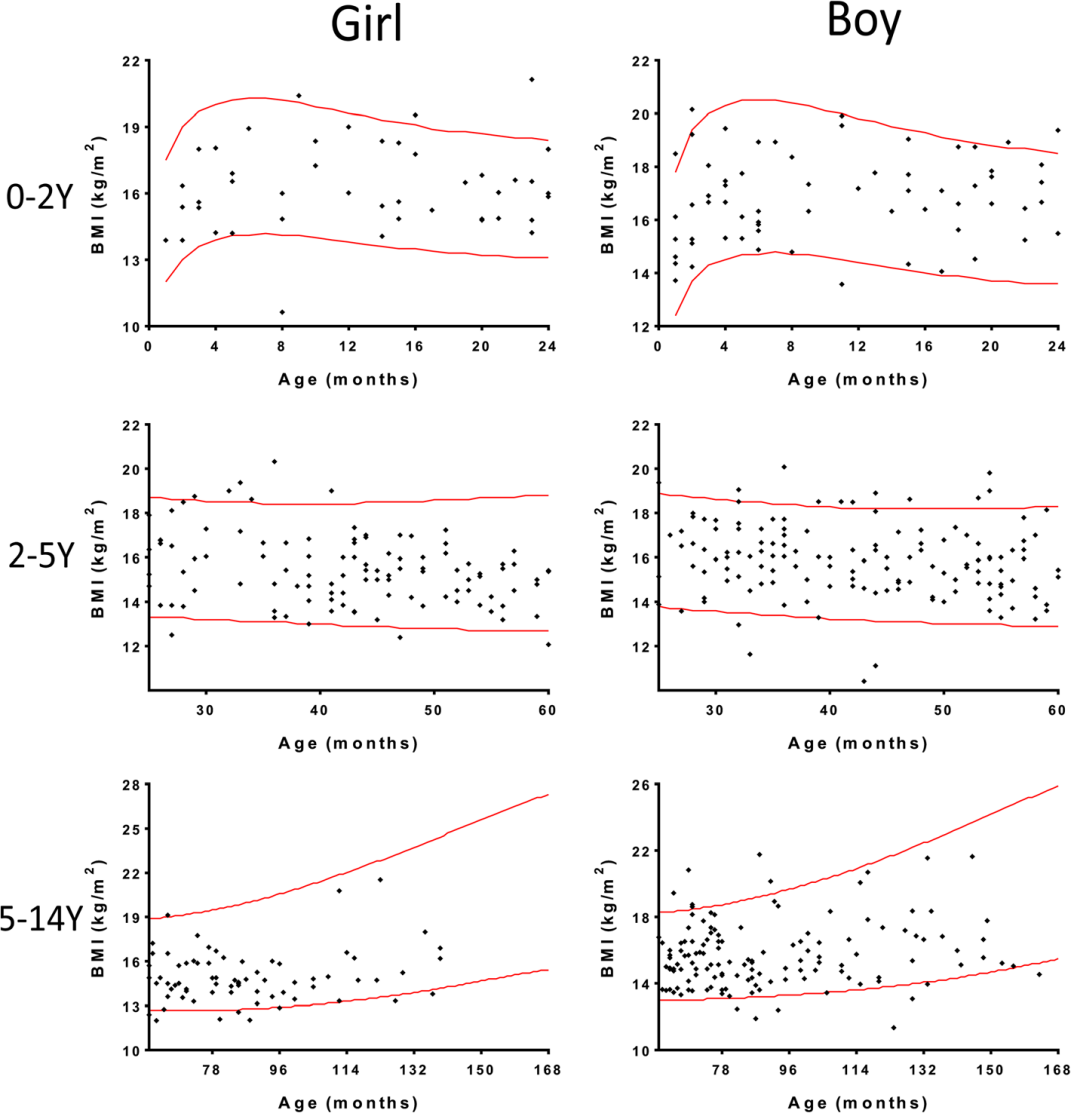
Distribution of the BMIs of all children with CC by gender and age. Most of the BMI measurements were within the normal range (red line: ±95% confidence interval of the WHO Reference). BMI: body mass index; CC: congenital cataract; Y: years

The distribution of the height, weight, and BMI measurements of all children with CC are shown in the scatterplots in Figs. 3, 4 and 5, respectively. According to the semi-quantitative analysis, the measurements of height, weight, and BMI of most children with CC were within the normal ranges (±95% confidence interval [CI] of the WHO Reference).

For further comparison with the WHO Reference values, the parameters of physical development of the children with CC were averaged for every month of age and then compared with the WHO Reference values after age matching. As shown in Tables 1 and 2, the heights of both girls and boys aged 2–5 years were shorter, and the girls aged 5–14 years exhibited a shorter height and a lower body weight and BMI than the WHO Reference values.

**Table 1:** Comparisons of the height, weight and BMI of the CC girls to the WHO Child Growth Standards

Girl		0-2Y *n* = 20	2-5Y *n* = 32	5-14Y *n* = 43 (36 in weight)
Height	CC	74.47 ± 10.14	97.22 ± 6.52	122.55 ± 11.79
(cm)	Standards	73.17 ± 10.12	98.70 ± 6.83	124.61 ± 11.44
	t	1.54	−0.50	−2.66
	P	0.139	**0.018**	**0.011**
Weight	CC	9.34 ± 2.37	14.62 ± 2.21	20.84 ± 3.81
(kg)	Standards	8.69 ± 2.16	14.97 ± 1.95	22.77 ± 3.69
	t	3.91	−0.84	−4.85
	P	**0.001**	0.408	**<0.001**
BMI	CC	16.68 ± 1.99	15.61 ± 1.43	14.98 ± 1.65
(kg/m^2^)	Standards	16.03 ± 0.62	15.37 ± 0.13	15.79 ± 0.72
	t	1.65	1.00	−3.42
	P	0.116	0.325	**0.001**

Notes: Nine children with outliers (out of mean ± 3 SD) were excluded in the measurements. Bold data are significant at *P* < 0.05

*Y* years, *CC* congenital cataract, *BMI* body mass index

**Table 2:** Comparisons of the height, weight and BMI of the CC boys to the WHO Child Growth Standards

Boy		0-2Y *n* = 24	2-5Y *n* = 36	5-14Y *n* = 63 (36 in weight)
Height	CC	75.31 ± 10.54	97.87 ± 7.79	128.65 ± 14.75
(cm)	Standards	74.92 ± 9.39	99.74 ± 6.50	129.14 ± 13.17
	t	0.34	−3.23	−0.51
	P	0.737	**0.003**	0.615
Weight	CC	9.82 ± 2.70	15.27 ± 2.19	23.45 ± 5.47
(kg)	Standards	9.38 ± 2.07	15.40 ± 1.79	23.53 ± 3.57
	t	1.42	−0.64	−0.12
	P	0.170	0.529	0.906
BMI	CC	16.89 ± 0.91	17.70 ± 10.34	15.85 ± 1.62
(kg/m^2^)	Standards	16.50 ± 0.65	15.49 ± 0.24	16.07 ± 0.88
	t	1.66	1.29	−1.06
	P	0.110	0.206	0.295

Notes: Eight children with outliers (out of the mean ± 3 SD) were excluded in the measurements. Bold data are significant at *P* < 0.05

*Y* years, *CC* congenital cataract, *BMI* body mass index

The children with CC and concomitant systemic diseases and those with a family history of CC were also further analyzed and compared with the WHO Reference. The heights of the children with CC and systemic diseases were shorter than the WHO Reference values (Table 3). The children with CC and a family history of the disease also exhibited shorter heights and lower BMIs than the children with CC without family histories and the WHO Reference values (Table 4).

**Table 3:** Comparisons of the height, weight and BMI measurements of the children with CC with/without systemic diseases against the WHO Reference

*N* = 40	CC + systemic disease	CC Control	Standards
Height	96.00 ± 22.26	98.48 ± 24.79	99.07 ± 23.87
(cm)	t	−1.72	−3.21
	P	0.094	**0.003**
Weight	15.71 ± 7.25	16.93 ± 8.15	16.23 ± 7.44
(kg)	t	−1.43	−0.82
	P	0.162	0.418
BMI	16.38 ± 3.94	16.88 ± 3.52	15.82 ± 0.72
(kg/m^2^)	t	−0.59	0.89
	P	0.556	0.381

Notes: Children with family histories were excluded in the measurements. Bold data are significant at *P* < 0.05

*Y* years, *CC* congenital cataract, *BMI* body mass index

**Table 4:** Comparisons of the height, weight, and BMI measurements of the children with CC with/without family histories against the WHO Reference

*N* = 124	CC+ family histories	CC Control	Standards
Height	99.80 ± 21.30	102.15 ± 22.09	103.04 ± 21.69
(cm)	t	−3.14	−5.39
	P	**0.002**	**<0.001**
Weight	15.58 ± 6.04	15.75 ± 5.80	15.98 ± 5.46
(kg)	t	−0.386	−1.231
*N* = 116	P	0.700	0.221
BMI	16.42 ± 3.60	15.69 ± 2.10	15.76 ± 0.70
(kg/m^2^)	t	2.02	2.04
	P	**0.045**	**0.043**

Notes: Children complicated with systemic diseases were excluded in the measurements. Bold data are significant at *P* < 0.05

*Y* years, *CC* congenital cataract, *BMI* body mass index

## Discussion

Height, weight, and BMI are the most important indexes of the physical development and nutrition status of children [6]. However, the distribution of the physical development indexes of children presenting with CC is still unclear, and nearly all published reports have focused on the diagnosis and treatment of the disease. This prospective study with a relatively large sample size is the first to summarize the height, weight, and BMI of children with CC. In the semi-quantitative analysis of scatterplots, the distributions of the height, weight, and BMI of most children with CC were within normal ranges according to the WHO Reference. However, in further comparisons after matching by age and gender, we found that the heights of the 2- to 5-year-old girls and boys were shorter, and the girls aged 5–14 years exhibited shorter heights as well as lower body weights and BMIs than the WHO Reference values.

CC can be divided into hereditary, non-hereditary and idiopathic based on etiology. Nearly 3/4 of children in this study had idiopathic and isolated cataracts, a localized ocular disorder that may not affect the general physical conditions, and thus the overall distributions of the height, weight, and BMI of most of the children with CC were within normal ranges. By contrast, hereditary CCs are often associated with systemic diseases that may affect the physical development and nutritional condition. Therefore, the children with CC and systemic diseases and those with a family history of CC in this study were also further analyzed. Among all of the children with CC, 6.72% (40/595) were complicated with systemic diseases, and 21.01% had a family history of the disease. The prevalence of concomitant systemic abnormalities in this study was lower than the findings of previous studies [5, 10], possibly due to the difference in standards and the limitation of specialized eye hospitals. However, the rate of a family history recorded in this study was similar to that in a Japanese investigation (21.01% vs. 22.4%) [11]. The results also showed that the children with CC with both systemic diseases and family histories had shorter heights and lower BMIs than either those without systemic diseases and family histories or the WHO Reference. Favism and congenital heart disease are the two most common concomitant systemic diseases in children with CC. Shorter height, lower weight, and malnutrition in children with congenital heart disease were also previously reported in 2015 by Hassan et al. [12]. The malnutrition of children with CC may result from chronic heart and lung dysfunction, from the metabolic disorders caused by favism [13], or from other concomitant systemic disorders. The physical development of the children with family histories may be indirectly affected for the following reasons: First, all of the children with CC and family histories included in this study exhibited bilateral involvement, and a long period of poor visual acuity limits outdoor exercise among children with CC, leading to a shorter height and lower BMI. Second, most parents of the children with a family history of CC are also cataract sufferers, and they are unable to adequately care for their children because of low vision. Low parental education levels and low-income economies [14] are factors that were previously reviewed in CC families, due to the poor vision caused by CC. In this study, we also investigated parental education levels and family incomes among children with CC; the results showed that less than 1/4 of mothers were highly educated, and more than half of the CC families had a family income below the city average. For children with CC with systemic diseases and family histories, physical strength training, nutrition education, and regular pediatric clinic visits are necessary, and political support from the government regarding education and economic conditions must also be emphasized.

Several factors may limit the extent to which this study’s results can be generalized. First, the prevalence of concomitant systemic diseases in children with CC may be under-estimated due to the limitation of specialized eye hospitals. Although we spared no efforts to confirm the general conditions of the children with CC by routine physical examinations, medical records, and parent statements, some indistinguishable systemic diseases might have been omitted. Second, the results of this study remain limited to one eye-care facility in China, and further multi-center studies are required to more clearly elucidate the physical development of children with CC in other eye hospitals located in different districts and with different levels of medical care.

## Conclusions

Despite its limitations, this study is the first investigation to summarize the physical development status of children with untreated CC. The height, weight, and BMI measurements of most of the investigated children with CC were within the normal ranges of the WHO Reference. However, the children with CC with concomitant systemic diseases and those with family histories exhibited shorter heights and lower BMIs. The information regarding height, weight, and BMI helps in understanding the physical development and nutritional condition of children with CC, and the findings of this study may provide a useful reference for national management strategies to diagnose and treat CC.

## Abbreviations

BMI: Body mass index
CC: Congenital cataracts
CI: Confidence interval
CNY: China Yuan
mean ± SD: the mean and standard deviation
WHO Reference: the World Health Organization Child Growth Reference
Y: Years
ZOC: Zhongshan Ophthalmic Center
